# The Piggy Solution: Harnessing Food Waste for Sustainable Hog Farming

**DOI:** 10.1002/gch2.202500073

**Published:** 2025-07-16

**Authors:** Matthew C. Ogwu, Catherine E. Hills, Silvana Pietrosemoli

**Affiliations:** ^1^ Goodnight Family Department of Sustainable Development Appalachian State University Living Learning Center 305 Bodenheimer Drive Boone NC 28608 USA; ^2^ Alternative Swine Research and Extension Project Department of Animal Science 319A Polk Hall, Campus Box 7621 Raleigh NC 27696 USA

**Keywords:** animal feed, feed efficiency, food wastes, hog farming, waste management

## Abstract

The increasing global population and concomitant rise in food demand lead to significant challenges in sustainable agricultural practices and food waste management. This review explores a promising solution to these challenges by examining the potential of utilizing food waste in hog farming as a sustainable feed resource. The paper highlights the environmental, economic, and social benefits of diverting food waste from landfills and repurposing it for livestock nutrition. Nutritional adequacy, safety, and regulatory frameworks surrounding the use of food waste in hog diets, as well as technological advancements and logistical considerations necessary for the widespread adoption of this practice, are discussed along with pilot projects that have successfully implemented food waste feeding programs, assessing their outcomes in terms of feed efficiency, animal health, and environmental impact. Using food waste as animal feed provides a cost‐effective alternative to traditional feedstuffs. It also contributes to the global goal of reducing the food, land, and greenhouse gas (GHG) mitigation gaps by 12%, 27%, and 15%, respectively, by 2050. This practice will significantly lower the carbon footprint of hog farming by redirecting 45% of GHG emissions from conventional feed production to promote a circular economy within the agricultural sector. However, successfully implementing food waste feeding programs requires stringent monitoring and adherence to safety standards to prevent contamination and ensure animal welfare.

## Graphical abstract

This review explores the use of food waste as a sustainable feed alternative for hog farming. By repurposing food waste, the approach reduces landfill waste, lowers feed costs, and mitigates environmental impacts like greenhouse gas emissions. The study discusses nutritional adequacy, safety regulations, processing methods, and business models essential for the successful large‐scale implementation of food‐waste‐to‐feed systems.

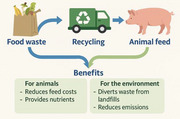

## 1 Introduction

In contemporary times, the sustainable utilization of food and allied resources has become paramount in addressing global challenges such as food security and environmental degradation.^[^ ^1^, ^2^ ^]^ Food security, environmental sustainability, resource conservation, and the issue of food waste (FW) are among global critical challenges. Food waste is a critical issue with far‐reaching consequences.^[^ ^3^ ^]^ It is increasingly recognized as a wicked problem, characterized by its complex, interdependent causes and far‐reaching social, economic, and environmental implications. The food category with the most significant amount of waste among household FW is fruits and vegetables, followed by poultry, meat, eggs, and dairy products, whereas crop straw, livestock, and poultry manure are the largest sources of agricultural waste from grain‐producer countries.^[^ ^4^, ^5^ ^]^ China, the United States, and India produce more household FW than any other country, with an estimated 92 million, 80 million, and 69 million metric tons yearly, respectively.^[^ ^6^, ^7^, ^8^, ^9^ ^]^ This wastage represents a significant loss of resources and contributes to environmental degradation and greenhouse gas emissions, exacerbating food insecurity. However, amidst these challenges, innovative solutions are emerging to address the problem of FW and its associated impacts.

On the other hand, the populace of many countries, especially those in the Global South, suffers from chronic food insecurity.^[^ ^10^ ^]^ Just because FW are sometimes considered no longer viable for safe human consumption does not mean they are worthless. All that “waste” is still very dense in nutrients that only become useless once deposited into a landfill. When food waste ends up in landfills, it decomposes anaerobically, producing methane—a greenhouse gas with a global warming potential ≈28 times greater than carbon dioxide over a 100‐year period.^[^ ^11^, ^12^ ^]^ Additionally, the leachate from decomposing organic waste can contaminate groundwater and surrounding ecosystems, posing further environmental risks.^[^ ^13^, ^14^ ^]^ Humanity faces unprecedented FW and limited options for mitigating it.^[^ ^15^, ^16^ ^]^ There is an over‐reliance on composting these resources while other viable options exist.^[^ ^17^ ^]^ Food waste can be collected and repurposed as a helpful diet resource for domesticated animals, especially pigs. Pigs are known to eat almost everything—the stalks from lettuce, the shells from your eggs, and the fruit that is still perfectly fine but has passed its sell‐by date (Chassé et al., 2019).^[^ ^18^, ^19^ ^]^ Based on available knowledge, pigs, like humans, are susceptible to digestive disturbances from spoiled food.^[^ ^20^ ^]^ Extant literature suggests that pigs, despite their susceptibility to viral and foodborne pathogens, typically maintain efficient digestive function.^[^ ^20^, ^21^ ^]^

Food waste occurs at various stages of the food system, including production, processing, distribution, and consumption. In developed countries, most FW originates from households, restaurants, and supermarkets. In contrast, in developing nations, losses predominantly occur during production and distribution due to inadequate infrastructure and post‐harvest handling practices. The environmental and socio‐economic consequences of FW are profound. It squanders valuable resources such as water, land, and energy and generates greenhouse gas emissions, primarily methane, when disposed of in landfills.^[^ ^22^, ^23^ ^]^ Moreover, FW exacerbates food insecurity by diverting resources away from those in need and contributes to higher food prices and market volatility.^[^ ^24^ ^]^

Sustainable hog farming represents a holistic approach to pork production that emphasizes environmental stewardship, animal welfare, and economic viability. Unlike conventional intensive farming methods characterized by concentrated animal feeding operations (CAFOs) and reliance on grain‐based feeds, sustainable hog farming prioritizes pasture‐based systems, diversified diets, and rotational grazing to promote soil health, biodiversity, and animal well‐being.^[^ ^25^, ^26^, ^27^, ^28^ ^]^ By incorporating principles of agroecology and regenerative agriculture, sustainable hog farming systems aim to minimize external inputs, reduce environmental impacts, and foster resilience against climate change and market fluctuations.^[^ ^29^, ^30^ ^]^ Additionally, these systems prioritize animal welfare by providing access to outdoor spaces and natural foraging opportunities and minimizing stress‐inducing practices such as overcrowding and confinement.^[^ ^31^, ^32^ ^]^

This review aims to discuss the concept of harnessing FW for sustainable hog farming, exploring its feasibility, implications, and potential impact on agriculture, the environment, and society. It will contribute toward understanding FW reduction strategies, particularly by feeding FW to hogs to reduce environmental footprint, improve animal welfare, and enhance the quality of pork products. At the intersection of FW management and sustainable hog farming lies the Piggy Solution – a holistic approach that utilizes FW as feed for pigs. Rather than discarding surplus food destined for landfill or composting, the Piggy Solution highlights the repurposing of FW as a valuable resource in hog farming operations. Unlike traditional FW‐to‐feed practices, which are often small‐scale, unregulated, or informal, the Piggy Solution represents a structured, integrative approach that emphasizes regulatory compliance, scalable processing technologies, and alignment with circular economy principles to ensure food safety and environmental sustainability. This concept embodies the principles of a circular economy, where waste is minimized and resources are efficiently utilized to create value across the agricultural value chain.^[^ ^33^, ^34^, ^35^ ^]^ It offers insights into a timeless solution for FW that holds the potential to address pressing global challenges while fostering resilience and sustainability in the farming sector.^[^ ^17^, ^34^, ^36^ ^]^

## 2 Food Waste: Causes, Composition, Consequences, and Impacts

**Figure 1:**
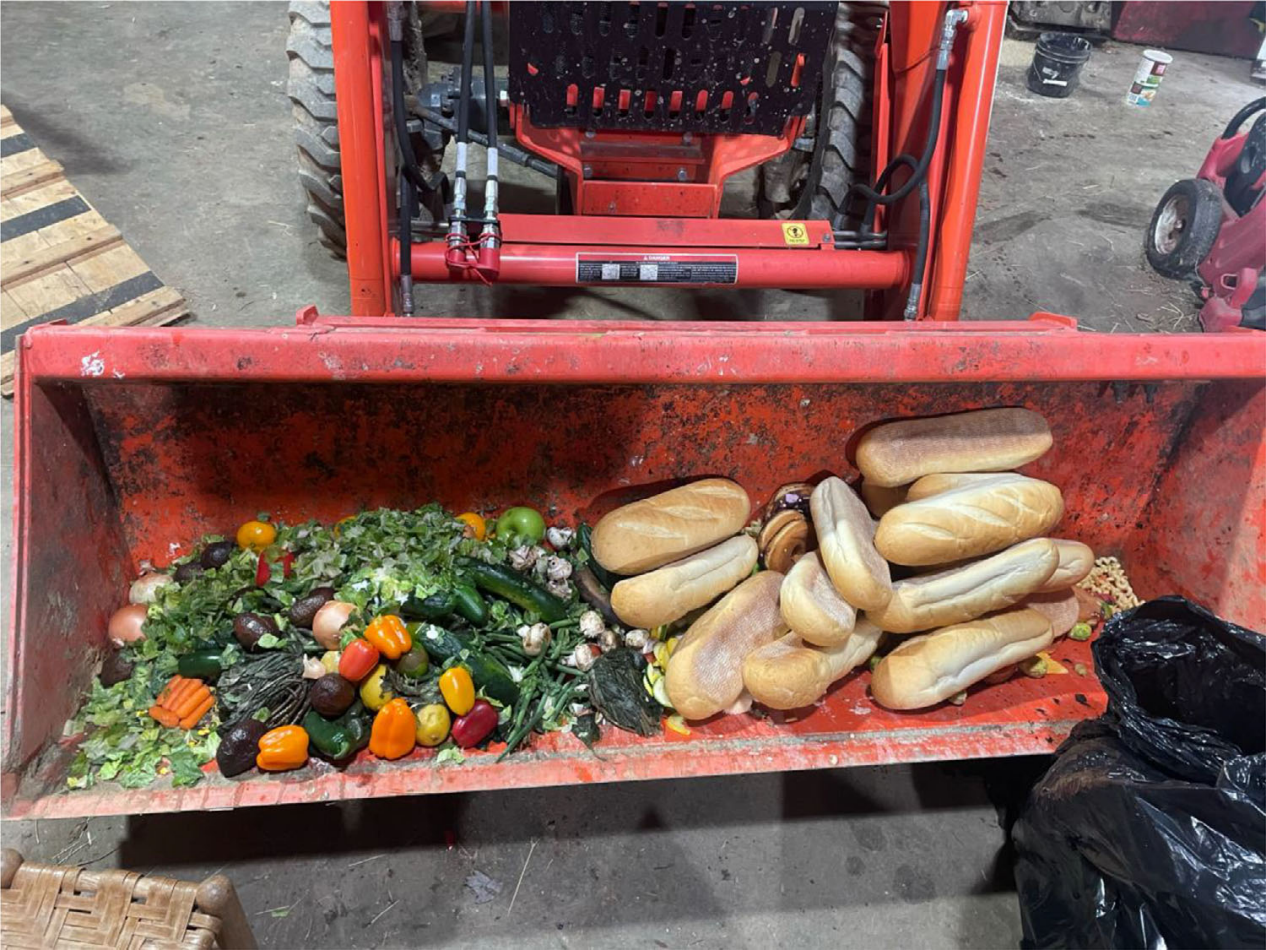
Food and agricultural scraps and waste collected in tractor loaders ready for processing. Photo Credit: C.E. Hills.

**Figure 2:**
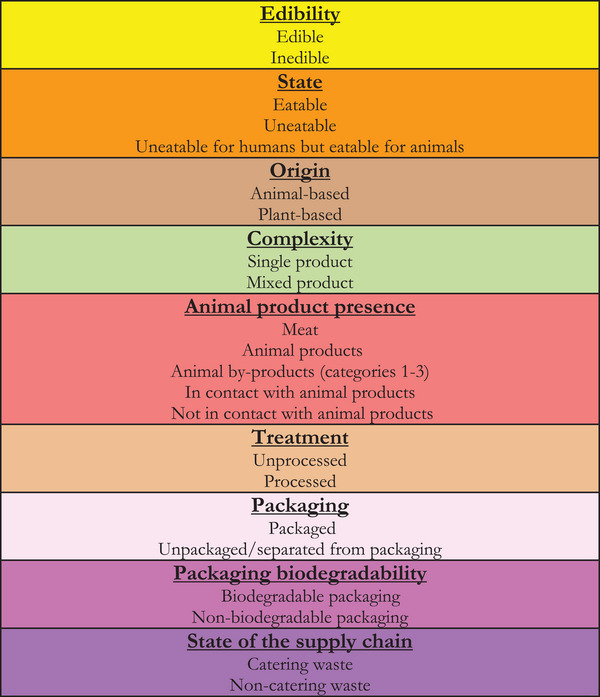
Categorization of food waste Source: Adapted from ref. [39, 40].

Food waste is a significant global challenge with profound environmental, economic, and social implications.^[^ ^22^ ^]^ Food waste encompasses discarded, lost, or uneaten food along the supply chain, including pre‐consumer waste at production, processing, distribution, and retail stages or post‐consumer waste from food services or households (**Figure** **1**). By‐product is the organic waste produced in the manufacturing or making of goods like inedible produce skins, animal fat, and other trimmings from food products before market. Types of FW include edible items discarded due to spoilage or expiration, trimmings and peels, surplus or excess food, and plate waste generated during meals. Food waste is primarily a product of overproduction, systemic inefficiencies, human behavior and attitudes, and bulk food purchasing worldwide.^[^ ^37^ ^]^ Understanding FW's composition, causes, consequences, and impacts is essential for developing effective strategies to mitigate its adverse effects and promote sustainability in the food system (**Figure** **2**).^[^ ^38^ ^]^

Food waste has seen a surge in recent decades, around the same period when a growing American preference to eat out has proven to be a large part of that. Oria and Schneeman^[^ ^41^ ^]^ cited the work of Gunders^[^ ^42^ ^]^ on “plate waste,” which found that restaurants produce copious FW with a significant increase since 1982 and with increased serving sizes that exceed the United States Food and Drug Administration standards, more food is wasted or consumed at unhealthy quantities. Although dining establishments make money, consumers often overpay, and 17% of meals are left uneaten.^[^ ^43^, ^44^ ^]^ This food usually continues down our linear food chain to a landfill where any value it still has post‐consumer officially becomes a waste.

The composition of FW varies depending on factors such as location, culture, and consumption patterns. The composition of FW can be broken down into biological, physical, and chemical components. The biological composition includes organic matter and microbes and macrobes. Food waste primarily consists of organic matter, such as carbohydrates, proteins, and fats, which can decompose and break down through biological processes. Food waste may contain various microorganisms, including bacteria, fungi, and insects, which play a role in the decomposition of organic matter.^[^ ^45^, ^46^ ^]^ Food waste can contain solids such as fruit and vegetable peelings, meat trimmings, bones, coffee grounds, and other non‐liquid components. Food waste may contain liquid components such as soups, sauces, and beverages. Interactions between FW and microorganisms produce diverse gases and bioactive materials.^[^ ^47^, ^48^ ^]^ Food waste includes fats and oils from cooking, dairy products, and processed foods like cooking oils, butter, and other leftover and discarded fats. The minerals and trace elements composition of FW is essential for plant and microbial growth, which can influence decomposition processes (ref. [49, 50] **Table** **1**).

**Table 1:** Classification, sources, and examples of different types of food waste across the supply chain.

Type of food waste	Explanation	Sources	Examples	Refs.
Agricultural waste	Food is discarded at the agricultural production stage due to factors such as overproduction, market demand fluctuations, or cosmetic imperfections.	Farms (during production and harvesting), storage, and processing facilities	Unharvested crops, surplus produce, rejected fruits/vegetables.	[51, 52]
Post‐harvest waste	Losses occurring during harvesting, handling, and transportation, often due to inadequate infrastructure or storage facilities.	Farms, storage facilities, transportation, and markets (Unsold produce, surplus inventory, or expired goods)	Spilled grains, bruised fruits, and damaged goods during transit.	[53, 54]
Retail waste	Food discarded by retailers due to expiration dates, damaged packaging, or overstocking.	Supermarkets/grocery stores, as well as restaurants/cafes and markets	Expired dairy products, unsold bakery items, and damaged goods.	[55, 212]
Consumer waste	Food is wasted by consumers at home, restaurants, or institutions, often due to overbuying, improper storage, or reluctance to consume leftovers.	Homes, restaurants/cafes, and institutions	Leftover meals, expired groceries, and untouched food at buffets.	[38, 56]
Economic factors	Perceived low value or high cost of recovering food waste, fluctuations in market prices or demand for certain foods, lack of financial incentives to minimize waste, such as disposal costs or tax penalties	Cost concerns, market prices, financial incentives, and minimal financial consequences for wasting food	Businesses choosing to discard food rather than invest in recovery, Surplus food sold at prices lower than production costs	[55, 212]

The omnivorous pig can eat a variety of feedstuffs. If things like meat are cooked and there is no mold on food, they can eat it. This includes all parts of fruits and vegetables, bakery and cereal waste, dairy, and restaurant and household waste. The challenging parts surrounding what is legally known as “garbage feeding” come from the legal side of things; many health acts, regulations, and laws have been put in place to prevent unhealthy feeding practices and transmittable diseases for the sake of pigs and people. For example, in North Carolina (USA), if FW is only being collected from sources without contact with meat, fish, or poultry, no license is required to carry out this procedure. However, licensing and pasteurization are necessary to widen the scope of FW reduction and collect all offal food.^[^ ^57^ ^]^ In contrast, the European Union enforces stricter centralized regulations under EC No. 1774/2002, which prohibits the use of catering waste containing meat or animal by‐products in animal feed due to concerns over diseases such as foot‐and‐mouth.^[^ ^58^, ^59^, ^60^ ^]^ Former foodstuffs that have not been in contact with meat may be allowed but must meet traceability and hygiene criteria. These rules reflect a precautionary approach and prioritize public and animal health within a unified regulatory framework. In Asia, regulatory diversity is more pronounced. For example,^[^ ^15^ ^]^ describe how China has adopted pilot programs in several provinces that allow food waste recycling into animal feed under tightly monitored conditions, including heat treatment and licensing. Japan, meanwhile, has established a more formalized system known as Eco‐feed, which permits FW feeding to livestock if it meets strict safety and quality controls through sterilization and nutrient balancing.

Causes of FW include inefficiencies in production and distribution, overproduction and over‐purchasing, aesthetic standards, improper storage and handling, lack of education surrounding proper waste disposal and management, and consumer behaviors such as confusion over date labels and portion sizes.^[^ ^52^, ^55^ ^]^ Consequences of FW encompass economic losses, resource depletion, greenhouse gas emissions, biodiversity loss, and social inequities. Food waste has significant environmental impacts, contributing to greenhouse gas emissions, water and land use, and biodiversity loss.^[^ ^61^ ^]^ For instance, offal food deposited in places like landfills undergoes an anaerobic reaction and releases methane. This greenhouse gas has a much higher global warming potential than carbon dioxide. Economically, FW represents a loss of resources throughout the supply chain, including labor, energy, and capital investments. Socially, FW exacerbates food insecurity, perpetuates inequalities, and undermines efforts to achieve sustainable development goals.^[^ ^62^ ^]^ Swine feed is often made up of dried crops like soy or corn, and with biofuels taking off worldwide, especially in the U.S., feed providers have sought new origins for these grains. The transnational grain trade has consequently been picking up speed and is accelerating deforestation in places like the Amazon rainforest in Brazil, offering few local economic improvements.^[^ ^63^ ^]^ The transnational grain trade is also interrupting our planet's nitrogen cycle through the “nitrogen cascade effect,” which happens when plants like the soybean, which may have been grown in Brazil, cannot redistribute their nutrient contents locally. Instead, it is removed and taken somewhere else, creating an imbalance, a deficiency that will require fertilizers at the origin and generate an excess of nutrients at the final destination.^[^ ^64^ ^]^

## 3 Pig Farming: Challenges and Feeding Strategies

**Figure 3:**
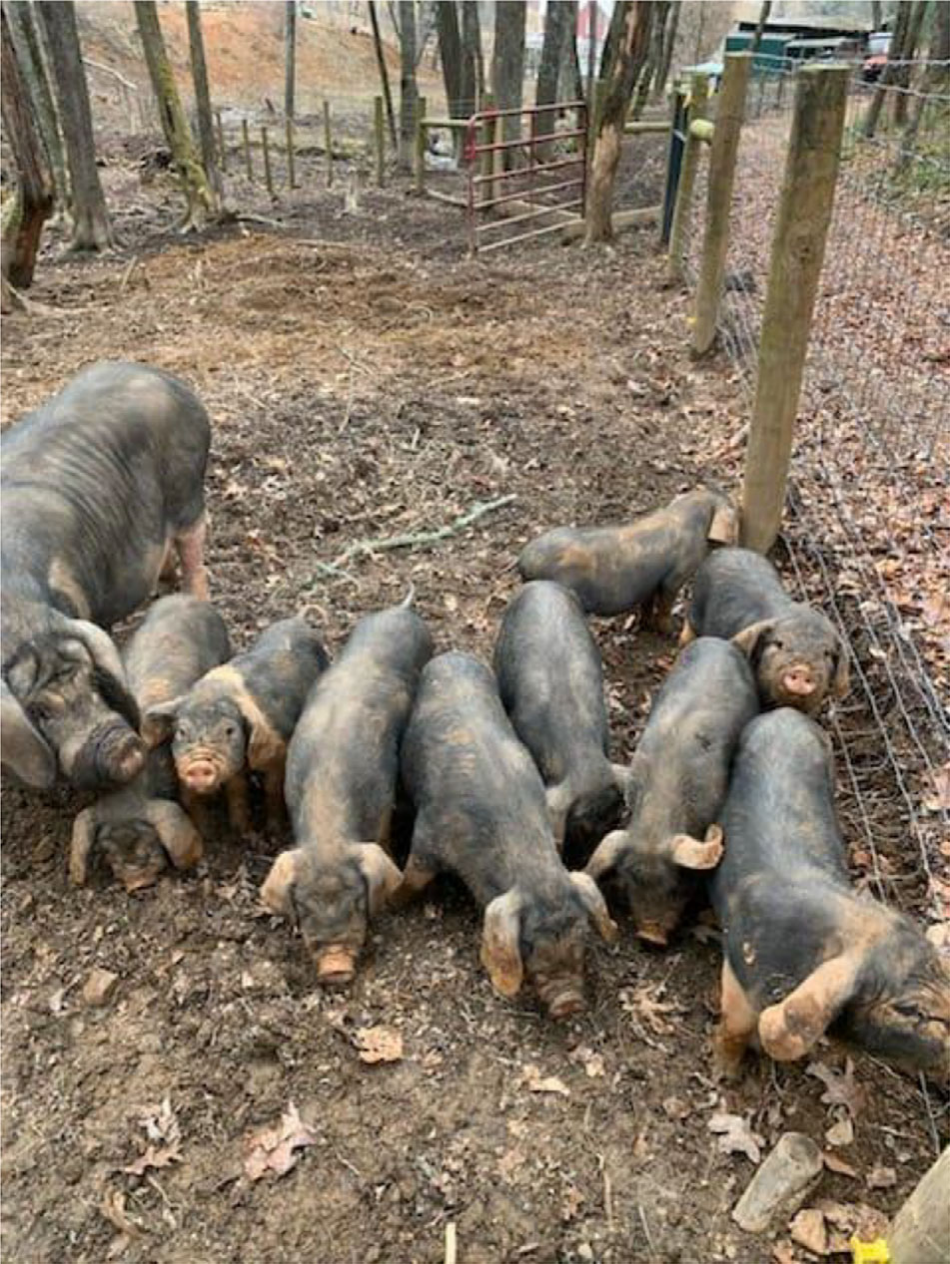
A group of pigs in a farm in a woodland environment. Photo Credit: C.E. Hills.

Pigs (*Sus scrofa domesticus*) are domesticated mammals from the Suidae family. Pigs were first domesticated between ≈8500–8000 BC around the present‐day Middle East (**Figure** **3**; ref. [65]). They are highly adaptable animals with diverse breeds, varying in size, color, and characteristics. Pigs have a complex digestive system like other monogastric animals, consisting of a single‐chambered stomach and a relatively simple large intestine.^[^ ^20^, ^66^ ^]^ Pigs are omnivores, and their diet includes grains, vegetables, fruits, and occasionally meat or animal by‐products. According to Sasaki and Koketsu,^[^ ^67^ ^]^ pigs have a relatively short gestation period of ≈3 months, 3 weeks, and 3 days (≈114 days). Sows typically give birth to litters of piglets, which they nurse for several weeks before weaning. Pigs are social animals that thrive in groups or herds. They exhibit complex social behaviors, hierarchy formation, and communication through vocalizations, body language, and olfactory cues.

Like any agricultural practice, hog farming comes with challenges, ranging from environmental concerns to animal welfare issues and economic constraints. Hog farming can have significant ecological impacts, including pollution of air, water, and soil.^[^ ^68^ ^]^ The release of manure and wastewater from large‐scale hog operations can contribute to water contamination of surface and groundwater, nutrient runoff, and the proliferation of harmful algal blooms because of their high nitrogen and phosphorus nutrient content. Runoff from hog farms can carry pathogens, antibiotics, and other pollutants into nearby water bodies, posing risks to aquatic ecosystems and human health and leading to ecosystem degradation.^[^ ^69^ ^]^ Additionally, emissions of greenhouse gasses such as methane and nitrous oxide from manure management and enteric fermentation can contribute to climate change.^[^ ^70^, ^71^ ^]^ To mitigate these impacts, various strategies have been employed, including anaerobic digestion, which captures methane for energy while reducing pathogen loads; constructed wetlands, which filter nutrients and contaminants from runoff; and vegetative buffer strips, which slow runoff and promote nutrient uptake before it reaches waterways.^[^ ^72^, ^73^, ^74^ ^]^

Diseases such as African swine fever, porcine epidemic diarrhea virus, and porcine reproductive and respiratory syndrome can devastate hog populations, leading to significant economic losses for farmers and disruptions to the pork supply chain.^[^ ^75^, ^76^ ^]^ The use of antibiotics in hog farming for disease prevention and growth promotion has raised concerns about the emergence of antibiotic‐resistant bacteria. Overuse and misuse of antibiotics in livestock production can contribute to the development of antibiotic‐resistant strains of bacteria, posing risks to public health by transmitting resistant pathogens to humans and compromising the effectiveness of antibiotics in treating human and animal infections. Economic factors such as fluctuating market prices, input costs, and regulatory compliance requirements can challenge the economic sustainability of hog farming operations.^[^ ^77^ ^]^ Small‐scale and family‐owned hog farms may face financial pressures due to competition from larger, industrialized operations and market volatility. Compliance with environmental regulations, zoning ordinances, and animal welfare standards can be challenging for hog farmers, notably smaller operations with limited resources. Meeting regulatory requirements for waste management, odor control, and water quality protection may require investments in infrastructure and technology upgrades.

Hog farming, particularly in the context of CAFO, presents several challenges, ranging from environmental concerns to animal welfare issues.^[^ ^78^, ^79^ ^]^ CAFOs often confine large numbers of hogs in crowded and restrictive conditions, limiting their ability to express natural behaviors, such as rooting and foraging.^[^ ^27^, ^79^ ^]^ They also produce massive amounts of biological waste, contributing to climate change and water pollution. Pigs raised in CAFOs are fed a diet consisting primarily of corn and soybeans, formulated to fit pigs’ nutritional requirements during the different stages of their life.^[^ ^80^ ^]^ The reliance on corn and soy as conventional feed sources contributes significantly to deforestation, water use, and greenhouse gas emissions. In contrast, recent life‐cycle assessments suggest that food FW‐based feed alternatives offer a more sustainable option. For example,^[^ ^81^ ^]^ found that using FW‐based feeds can reduce greenhouse gas emissions, land use, and water consumption compared to traditional corn‐soy diets. These findings underscore the environmental advantages of incorporating surplus or discarded food into animal feed systems. The practice will contribute to the Paris Agreement goal to reduce the food, land, and greenhouse gas mitigation gaps by 12%, 27%, and 15%, respectively by 2050. The continued heavy reliance on maize as swine feed was found to have the largest impact on global warming, freshwater eutrophication, and land usage in a case study that focused on the life cycle assessment of pigs fed alternative diets of things like rice and sorghum.^[^ ^82^ ^]^ Redirecting FW to supplement or replace a corn‐based hog diet in CAFOs would reduce their net carbon emissions and reduce overreliance on the mass production of corn. Sustainable hog farming relies on diverse feed sources to meet the nutritional needs of pigs while minimizing environmental impact. Familiar feed sources for pigs are included in **Table** **2** and include grains, protein sources, forages, food waste, etc.

**Table 2:** Feed categories for pigs: composition, nutritional roles, and sustainability benefits.

Feed category	Description and nutritional role	Refs.
Grains	Corn, wheat, barley, and sorghum are staple grains used in pig feed formulations due to their high energy content and digestibility.	[83, 84]
Protein sources	Soybean meal, canola meal, and sunflower meal are familiar protein sources used to supplement pig diets and provide essential amino acids.	[84, 85, 86]
Forages	Pasture and forage crops such as alfalfa, clover, and grasses can supplement pig diets, providing fiber, vitamins, and minerals.	[87, 88]
By‐products	Agricultural by‐products such as distillers' grains, rice bran, and wheat bran can be used as feed ingredients, reducing waste and improving feed efficiency.	[89, 90]
Food waste	Surplus or expired food products from supermarkets, restaurants, and food processing facilities can be repurposed as feed for pigs, contributing to waste reduction and resource utilization.	[17, 91]

To ensure sustainable hog farming operations encompass a range of practices aimed at promoting environmental stewardship, animal welfare, and economic viability, the raising methods represented in **Table** **3** must be implemented. These approaches reduce the environmental footprint of pig production and foster circularity through resource recycling, such as food waste and manure, contributing to a more resilient and regenerative agricultural system.

**Table 3:** Sustainable farm management practices enhancing food waste utilization in pig production.

Practice	Explanation and sustainability roles	Refs.
Rotational grazing	Allowing pigs access to pasture or rotational grazing systems promotes soil health, reduces erosion, and enhances nutrient cycling. When integrated with food waste utilization, such systems enable pigs to consume surplus organic matter while their manure enriches the soil—creating a closed‐loop, sustainable farming model.	[92]
Nutrient management	Implementing effective nutrient management plans helps minimize nutrient runoff and water pollution by optimizing manure application rates, timing, and methods.	[93]
Alternative housing systems	Providing pigs with access to outdoor environments, deep‐bedded pens, or hoop barns promotes natural behaviors, improves animal welfare, and reduces environmental impacts.	[94]
Integrated farming systems	Integrating pigs into diversified farming operations, such as agroforestry or mixed crop‐livestock systems, enhances resource efficiency, promotes biodiversity, and improves farm resilience. When coupled with food waste utilization, these systems enable farmers to repurpose organic waste as feed, reducing input costs and closing nutrient loops within the farm ecosystem.	[95]
Feed efficiency and optimization	Optimizing feed formulations, minimizing feed waste, and utilizing locally sourced feed ingredients reduce environmental footprint and production costs while ensuring optimal nutrition for pigs.	[96]

## 4 Processing Food Waste for Pig Consumption: Sustainable Pig Feeding Strategies and Techniques

**Figure 4:**
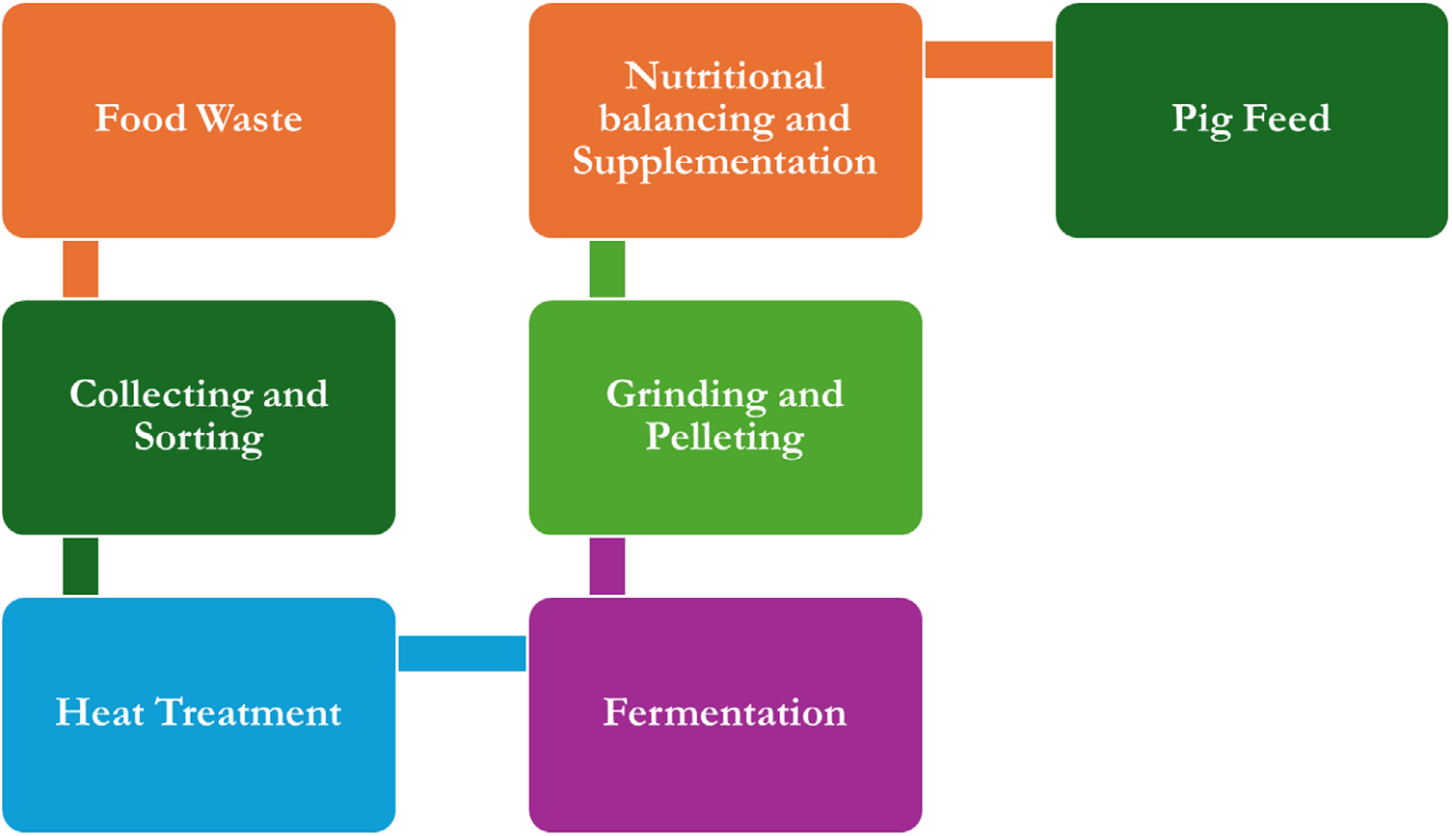
Food waste processing processes to pig feed. Credit: Authors.

Essential FW processing techniques for feeding FW to pigs include collecting and sorting the FW to remove contaminants and non‐edible materials (**Figure** **4**). Source separation at the point of generation (e.g., restaurants and households) improves the quality and safety of the collected waste. Encouraging source separation of FW at restaurants, cafeterias, and households enhances the quality and safety of the waste collected.^[^ ^97^, ^98^ ^]^ According to ref. [99], educating waste generators on proper separation techniques reduces contamination and enhances feed quality. After collection and sorting, the FW is subjected to heat treatment processes such as boiling, steaming, or autoclaving, essential to kill pathogens and ensure food safety. This step also helps to break down complex carbohydrates and proteins and kill non‐thermophilic microbes, making the waste more digestible for pigs.^[^ ^100^ ^]^ The heat‐treated FW may then be fermented. Fermentation can enhance the nutritional value of FW by increasing the availability of vitamins and amino acids.^[^ ^101^ ^]^ It also aids in preserving the waste, extending its shelf life, and reducing spoilage. The heat‐treated FW is ground into smaller particles to improve its digestibility and uniformity. Pelleting the processed waste facilitates storage, handling, and feeding, ensuring consistent intake by pigs. Supplementing processed FW with additional nutrients ensures a balanced diet for pigs. This may involve adding vitamins, minerals, and protein sources to meet the pigs' dietary requirements.

Currently, the swine industry faces significant challenges in achieving sustainability due to its impact on the environment, resource consumption, and animal welfare. Sustainable pig feeding strategies are essential for improving environmental stewardship, economic efficiency, and animal health. Sustainable pig feeding strategies aim to address these issues by optimizing feed efficiency, reducing waste, and enhancing the nutritional quality of feed.^[^ ^102^, ^103^ ^]^ Developing a sustainable pig‐feeding strategy will require the integration of nutritional science, waste management, and innovative technologies to create a holistic approach to pig farming (**Table** **4**).

**Table 4:** Essential considerations of nutritional optimization.

Strategy	Explanation and benefits	Refs.
Precision feeding	Precision feeding involves tailoring the diet to meet the specific nutritional requirements of individual pigs or groups based on age, weight, sex health, and physiological status. This approach reduces feed waste and improves nutrient utilization, improving growth performance and reducing environmental impact.	[96]
Alternative feed ingredients	Incorporating alternative feed ingredients, such as by‐products from the food and biofuel industries, can reduce reliance on conventional feed crops like corn and soybeans. Ingredients such as distillers, dried grains with solubles (DDGS), FW, and insect meal offer sustainable options that can lower feed costs and reduce the carbon footprint of pig farming.	Shursoon et al. (2022)[81]
Balanced diet formulation	Ensuring a balanced diet that meets the pigs' protein, energy, vitamins, and mineral requirements is crucial for optimizing growth and health. Feed additives such as enzymes, probiotics, and prebiotics can enhance nutrient digestibility and gut health, improving overall feed efficiency.	[104, 105]

In the context of waste management and resource efficiency, particular emphasis is placed on multiple interconnected sustainability strategies. These include optimizing feed utilization to reduce wastage, advancing manure management through renewable energy recovery and nutrient recycling, and enhancing water use efficiency via modern technological interventions (**Table** **5**).

**Table 5:** Waste management and resource efficiency.

Sustainability measure	Focus and benefits
Feed waste reduction	Implementing strategies to minimize feed wastage during storage, handling, and feeding can significantly improve sustainability. Techniques such as automated feeders, feed management software, and regular equipment maintenance help reduce spillage and spoilage.
Manure management	Efficient manure management practices are vital for reducing the environmental impact of pig farming. Technologies such as anaerobic digestion can convert manure into biogas, providing renewable energy and reducing greenhouse gas emissions. Additionally, manure can be processed into organic fertilizers, contributing to sustainable agriculture.
Water use efficiency	Optimizing water use in pig farming is essential for sustainability. Implementing water‐saving technologies, such as nipple drinkers and water recycling systems, can reduce water consumption and minimize the environmental footprint of pig production.

## 5 Feeding Food Waste to Pigs

**Figure 5:**
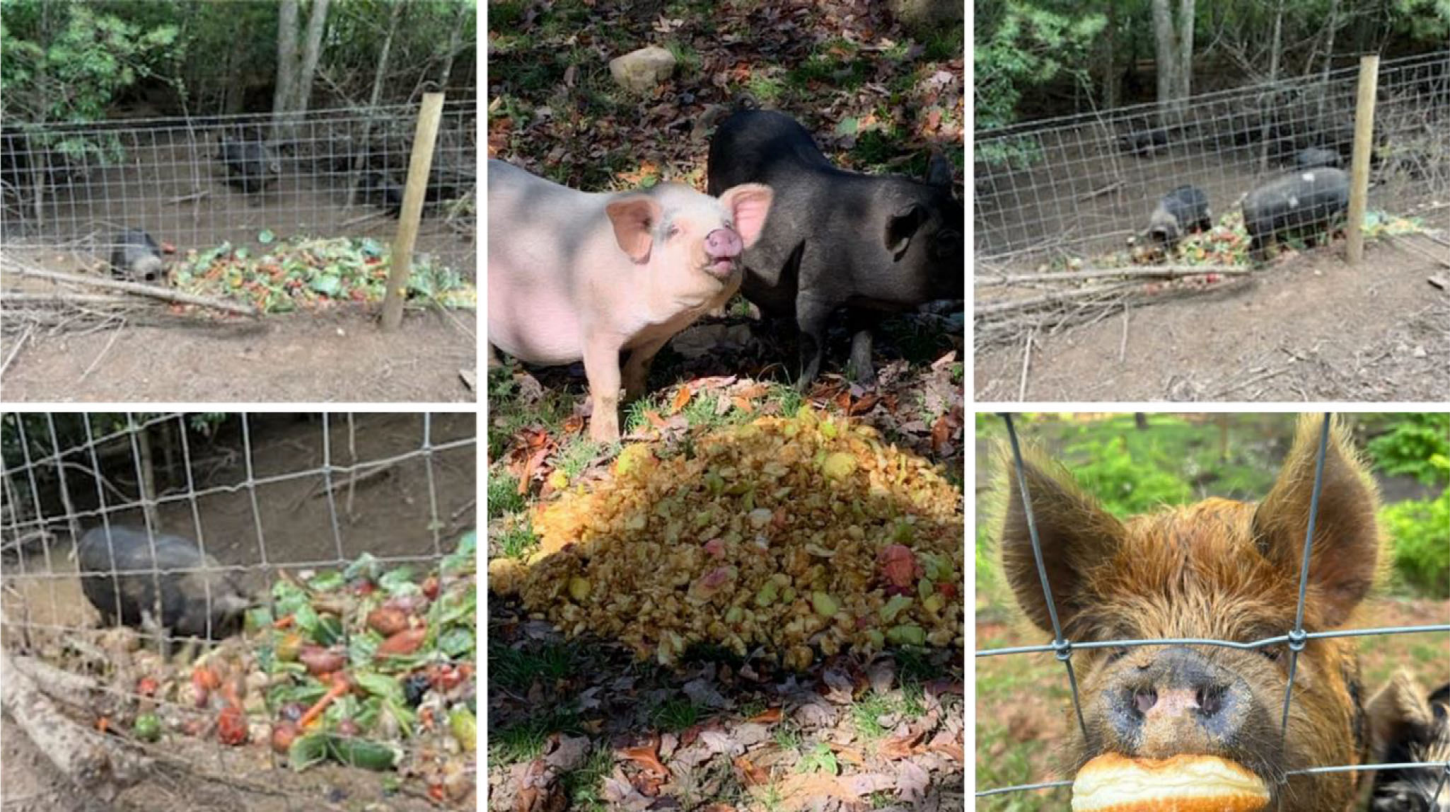
Utilization of food waste in sustainable swine farming as a recycling strategy for essential organic waste and nutrients like phosphorus. Photo Credit: C.E. Hills.

Feeding FW to pigs is a practice with benefits and potential risks. It helps to reduce the amount of food that ends up in landfills, which can contribute to environmental issues like methane production and soil contamination.^[^ ^17^, ^60^ ^]^ For instance, a famous Las Vegas livestock company collects FW from various sources, including local restaurants and casinos, and processes these scraps into livestock feed. These are then processed by separating the food from its packaging and pasteurizing the FW to ensure safety and quality (Combs Brothers LLC., n.d.). This method provides a sustainable feed source for their pigs and helps divert significant amounts of food waste from landfills. Utilizing FW as pig feed lowers the cost of raising pigs, as it provides an additional source of nutrition without the need to purchase commercial feed (**Figure** **5**). However, if implemented as illustrated in Figure 5, this practice may pose several biosecurity risks. These include cross‐contamination through the transfer of harmful microorganisms between surfaces, equipment, or environments, potentially leading to disease outbreaks; airborne transmission of pathogens carried by air currents, increasing the risk of infections spreading among animals and facilities; and the attraction of pests and wild animals such as predators, wild boars, feral pigs, and birds, which may act as vectors for disease or cause structural damage, further compromising farm biosecurity.

Nonetheless, feeding FW to domesticated pigs will make various nutrients accessible and available to the animals (ref. [18]; Sawyer et al.,^[^ ^215^ ^]^ 2023). Although FW is nutritious, it may not provide a balanced diet for pigs. Therefore, it could be a viable complete food or supplemented with commercial feed to a varied diet, essential to meet their nutritional needs. Regulations governing feeding FW to animals vary from state to state, including restrictions on certain types of waste or processing requirements to mitigate risks.^[^ ^15^ ^]^ There is a potential risk of disease transfer from food waste to pigs, which can impact humans through pork products if FW is not managed correctly. The work of ref. [106] summarizes the challenges that involve using diets with a greater/higher content of co‐products for pig feeding, such as the potential presence of antinutritional factors, incidence of mycotoxins and other possible contaminants, high fiber content, and variable nutritive value among batches (digestibility, protein and energy content). These factors can impact feed intake, animal performance (growth rate and feed efficiency), carcass quality, chemical and physical pork traits, and animal health and welfare. At the same time, these authors indicated the existence of knowledge and technology that can be used to overcome the above‐mentioned challenges. The study by ref. [107] examines the effects of enzymatically digested food waste on the fecal microbiota, meat quality, and carcass characteristics of growing‐finishing pigs. While including food waste in pig diets altered the fecal microbiota, suggesting potential health benefits for both pigs and humans, there were no observed differences in pork quality or carcass characteristics. This highlights the feasibility of using food waste as a sustainable feed alternative without compromising the final product. Márquez and Ramos^[^ ^108^ ^]^ demonstrated that diets incorporating food waste, such as fish and fruit by‐products, led to leaner carcasses and acceptable meat quality in pigs, suggesting early promise for FW‐based feeding strategies. More recently, Lestingi et al.^[^ ^109^ ^]^ conducted a comprehensive review showing that former food products can constitute up to 30% of pig diets without compromising growth performance, carcass characteristics, or meat quality. These findings affirm the viability of FW‐based feeds under modern nutritional balancing and regulatory standards, aligning with circular economy and sustainability goals. It is important to note that using fish waste leads to a slight fishy odor, and it highlights the importance of selecting appropriate food waste types to avoid undesirable sensory effects.

Regular monitoring of pigs' health for contaminants is needed to ensure their safety and strict adherence to regulations and guidelines regarding feeding practices. This ensures that livestock do not consume pesticides, heavy metals, or other pathogens.^[^ ^110^ ^]^ Farmers need to ensure that the FW being fed to their pigs comes from safe and reliable sources and is free from contaminants. The FW must be adequately processed through heat treatment (cooking) or fermentation to reduce the risk of pathogens and improve digestibility.^[^ ^111^ ^]^ These treatments would help improve nutrient availability and reduce antinutritional factors. The FW may also be supplemented by commercial feed to ensure that pigs receive a balanced diet that meets their nutritional requirements. Therefore, feeding FW to pigs can be a sustainable practice, but it requires careful planning and execution to ensure the health and safety of the animals.^[^ ^17^ ^]^ Some key strategies for effectively feeding FW to pigs while minimizing risks to animal health and welfare include:

### 5.1 Safe Source Selection for Food Waste

This refers to the choice of FW sources. Ensuring that the waste comes from reputable and safe sources, such as commercial kitchens, food processing plants, or farms, is imperative. This is necessary to avoid waste contaminated with chemicals or pathogens. Food waste from trusted organic and reputable suppliers is less likely to contain contaminants. Therefore, when selecting FW sources, it is essential to prioritize safety, quality, and nutritional value. Establish guidelines for acceptable types of FW and implement quality control measures to ensure the feed is safe and suitable for pigs.^[^ ^112^ ^]^ As a basic requirement, the FW must come from reputable establishments with good hygiene practices to minimize the risk of contamination.^[^ ^113^, ^114^ ^]^ Collaborations with urban food banks, supermarkets, school cafeterias, and restaurant chains can provide consistent streams of surplus food.^[^ ^115^ ^]^ Additionally, implementing traceability systems and adhering to organic waste handling standards such as the U.S. EPA's Food Recovery Hierarchy or the European Union's End‐of‐Waste criteria can enhance safety, legal compliance, and public trust.^[^ ^116^, ^117^ ^]^ Certifications such as ISO 14 001 for environmental management may also facilitate acceptance and integration of FW into circular agricultural models.^[^ ^118^ ^]^ Some avenues to source FW for swine feeding include commercial kitchens, grocery stores, wholesale markets, and others (**Table** **6**).

**Table 6:** Major food waste sources for swine feed with their nutritional potential, and sustainability contributions.

Source category	Relevance to hog feeding operation	Refs.
Commercial kitchens and restaurants	Generate large volumes of pre‐ and post‐consumer FW, often rich in fats, carbohydrates, and proteins; potential for direct use after proper treatment.	[119, 120]
Food processing facilities	Produce consistent, bulk quantities of organic by‐products such as peels, trimmings, and expired batches that can be safely repurposed into feed with minimal processing.	[60, 121]
Grocery Stores and Markets	Discard overripe, damaged, or unsold food products, including fruits, vegetables, and bakery items, suitable for inclusion in swine diets.	[122, 123]
Wholesale markets	Provide bulk quantities of unsellable produce and packaged goods that are still nutritionally valuable, enabling efficient feed sourcing.	[124, 125]
Food distribution centers	Contribute surplus and mispackaged food items that are safe but not viable for retail, offering a consistent supply stream for large operations.	[126, 127]
Food service providers	Institutions like schools and hospitals generate substantial leftover food suitable for recycling into animal feed after safety screening.	[119, 128]
Community food recovery programs	Collect excess edible food for redistribution; non‐redistributed portions can be diverted to animal feed, reducing landfill use.	[129, 130]
Local farms and orchards	Offer crop residues, culled produce, and spoiled harvests, supporting a circular economy within local agricultural systems.	[51, 119]
Food waste recycling companies	Specializing in collecting and processing food waste, ensuring compliance with health regulations and converting organic waste into standardized animal feed products.	[60, 93]
Food banks and charitable organizations	Redirect food surpluses to communities; non‐distributable portions are diverted to livestock feed, maximizing use and minimizing waste.	[123]

Establishments such as restaurants, cafeterias, and hotels generate significant amounts of FW daily. Food processing plants and manufacturing facilities generate byproducts during food production.^[^ ^131^ ^]^ Supermarkets, grocery stores, and farmers' markets often discard unsold or damaged products. Distribution centers produce large amounts of surplus or expired FW. Catering companies, event venues, and institutional food service providers generate FW from meal preparation and events. Most communities have food recovery programs and food banks that collect donations and surplus food from businesses, events, and households to redistribute to those in need but still end up with leftover FW (Lohnes 2021). Farms and orchards may have excess or damaged produce that is unsuitable for sale but still edible and nutritious. Companies specializing in FW recycling or composting could offer processed FW suitable for animal feed. An alternative would be wholesale markets where traded products may provide opportunities to obtain bulk quantities of FW at competitive prices.^[^ ^132^ ^]^ All these sources provide grains, fruit and vegetable trimmings, leftover cooked food, baked goods, proteins, and by‐products that have potential. The selection of food waste producers is endless.

### 5.2 Freshness of Food Waste

Ensuring the freshness of FW for hog feeding is essential for maintaining the quality and safety of the feed. Approaches for ensuring the freshness of FW are represented in **Table** **7**. Fresh FW is more likely to retain its nutritional value and palatability, enhancing its suitability as pig feed. Avoid waste that shows signs of mold, rot, or foul odors, as these may indicate contamination.^[^ ^81^, ^133^ ^]^ Regular monitoring and adjustments to the feeding program can help maintain high freshness and quality standards over time. The importance of using fresh FW in hog feeding programs includes nutritional value, palatability, and reduced risk of contamination (**Table** **8**). These approaches can be used to maintain the freshness of FW for hog‐feeding operations.

**Table 7:** Approaches for ensuring the freshness of FW used in hog feeding programs.

Practice	Explanation	Refs.
Regular inspection	Inspect food waste regularly for signs of spoilage, such as mold, off odors, or discoloration. Discard any waste that appears spoiled or unfit for consumption by hogs.	[91, 133]
Prompt collection	Collect food waste promptly from sources to minimize exposure to air and moisture, which can accelerate spoilage. Transport the waste to hog‐feeding areas as quickly as possible.	[134, 135]
Proper storage	Store food waste in appropriate containers or bins to maintain freshness and prevent contamination. Keep the waste covered and protect it from pests, sunlight, and other sources of contamination.	[37, 133]
Temperature control	Maintain proper temperature conditions during storage and transportation to slow down the rate of spoilage. Avoid exposing food waste to temperature extremes that can promote bacterial growth or enzyme activity.	[136, 137]
Rotation system	Implement a rotation system for food waste storage and usage to ensure that older waste is used first, minimizing the risk of spoilage and waste accumulation.	[138, 139]
Quality control	Establish quality control measures to monitor the freshness and safety of food waste, regularly conduct visual inspections, smell tests, and occasional sampling for microbial analysis to verify freshness.	[140, 141, 142]
Communication with suppliers	Maintain open communication with food waste suppliers to ensure that only fresh and suitable waste is provided for hog feeding. Address any concerns or issues regarding the quality of the waste promptly.	[91, 139]

**Table 8:** Criteria for defining the freshness of food waste.

Criteria	Benefit	Refs.
Nutritional value	Fresh FW contains higher levels of nutrients, including vitamins, minerals, and proteins, which are beneficial for the health and growth of hogs.	[37, 143]
Palatability	Fresh FW is more appetizing for hogs, leading to better acceptance and consumption. Stale or spoiled food may be rejected by hogs, resulting in waste.	[144]
Reduced risk of contamination	Fresh FW is less likely to harbor harmful bacteria, molds, or toxins compared to spoiled or expired food. Minimizing contamination risk is crucial for preventing illness in hogs.	[133]

### 5.3 Diverse Sources of Food Waste

Hog farmers should aim to obtain FW from various sources to provide a diverse diet for the pigs. Consider collecting from multiple FW producers to increase nutrient diversity through a wide selection of foods. Incorporating various sources of FW into hog feeding programs can provide a balanced and nutritious diet for the pigs while reducing waste. Sources of FW commonly used in hog feeding programs are presented in **Table** **9**. Most importantly, pig farmers should opt for FW sources that are locally available to reduce transportation costs and carbon footprint. Local sources may also offer fresher waste and foster community relationships. Explore partnerships with nearby businesses and organizations to obtain FW efficiently.

**Table 9:** Sources of food waste commonly used in hog feeding programs.

Food waste category	Feed characteristics and source details	Refs.
Fruits and vegetables	Surplus or unsold fruits and vegetables from grocery stores, markets, and Farms are excellent sources of food waste for hogs. These may include trimmings, peels, cores, and damaged produce.	[91]
Bakery waste	Bakery waste, such as stale bread, pastries, and unsold baked goods, can be repurposed as hog feed. These items are typically carbohydrate‐rich and may contain fat and proteins.	[145]
Grains and cereals	Excess or outdated grains and cereals from food processing or grain storage facilities can be used as hog feed. Examples include rice, oats, wheat, barley, and corn.	[146]
Dairy products	Surplus or expired dairy products, such as milk, cheese, yogurt, and whey, can be used as supplementary feed.	[147]
Meat and seafood by‐products	Meat trimmings, bones, and seafood by‐products from butcher shops, fish markets, and processing plants can be included in hog diets. These items provide valuable protein and minerals.	[131]
Restaurant scraps	Leftover food waste from restaurants, cafeterias, and catering events can be collected and used as hog feed. These may include cooked meats, vegetable trimmings, and leftover grains.	[148]
Grocery store waste	Unsold or damaged packaged food items, canned goods, and deli products from grocery stores can be diverted from landfills and fed to hogs; these items may include expired or near‐expiration products that are still safe for consumption by hogs.	[81, 139]
Food from food recovery programs	Participating in food recovery programs can provide access to a wide variety of surplus food items that are suitable for hog feed. These programs collect and redistribute surplus food from businesses, events, and households to minimize waste.	[81, 111]
Organic waste	Organic waste from certified organic sources, such as fruits, vegetables, grains, and dairy products, is generally safe for hog consumption and may offer additional health benefits.	[110, 111]

### 5.4 Nutritional Contents of the Food Waste

The nutritional content of the FW may be assessed to ensure it complements the pigs' dietary requirements. Food waste rich in carbohydrates, proteins, vitamins, and minerals can be valuable additions to the pigs' diet.^[^ ^149^ ^]^ Analyzing the nutritional composition of the waste and supplementing it with other feeds as needed will help to achieve a balanced diet. The nutritional content of FW used in hog feeding programs can vary widely depending on the types of food included. However, FW typically contains a combination of carbohydrates, proteins, fats, vitamins, and minerals, making it a valuable source of nutrients for pigs (**Table** **10**).

**Table 10:** Nutritional components of food waste and their functional benefits in swine diets.

Nutrient	Role and benefits	Refs.
Carbohydrates	FW such as grains, bread, fruits, and vegetables are rich in carbohydrates, providing pigs with energy for growth and activity.	[143]
Proteins	Protein‐rich FW sources include meat trimmings, dairy products, legumes, and certain grains. Proteins are essential for pigs' muscle development, immune function, and overall growth.	[150, 151]
Fats	Fats are found in FW items like meat trimmings, dairy products, and oily foods. While pigs require fats for energy, excessive fat consumption should be monitored to prevent obesity and related health issues.	[143, 152]
Vitamins	FW often contains vitamins such as A, C, D, and various B vitamins. These vitamins are crucial in pigs' metabolism, immune function, and overall health.	[143]
Minerals	Minerals like calcium, phosphorus, potassium, magnesium, and iron are present in FW from fruits, vegetables, grains, and meat products. These minerals are essential for pigs' bone development, muscle function, and other physiological processes.	[111, 143]
Fiber	Some FW items provide dietary fiber, particularly fruits, vegetables, and whole grains. Fiber aids in digestion and helps maintain gastrointestinal health in pigs.	[153]
Antioxidants and phytonutrients	Certain FW items, especially fruits and vegetables, contain antioxidants and phytonutrients that have health‐promoting properties. These compounds can help support the immune system and protect against oxidative stress.	[154]

It is important to note that while FW can provide valuable nutrients for pigs, it takes careful regulation to ensure they are receiving sufficient nutrients. Supplementing FW with commercial pig feed or other feed ingredients may ensure that pigs receive all the essential nutrients they need for optimal growth and health.^[^ ^155^ ^]^ Additionally, proper processing and handling of FW are critical to maintaining its nutritional integrity and preventing contamination. Regularly monitoring pigs' nutritional status and consulting with a veterinarian or nutritionist can help ensure that feeding programs meet the pigs' dietary requirements.

### 5.5 Contaminants in the Food Waste

Hog farmers should be vigilant about potential contaminants in FW, including chemicals, toxins, and pathogens. Avoid FW that may be contaminated with pesticides, cleaning chemicals, heavy metals, or harmful microorganisms, establish clear guidelines for acceptable contaminant levels, and conduct periodic testing to ensure compliance. Contaminants in FW used in hog feeding programs can pose risks to pig health and food safety if not properly managed.^[^ ^35^ ^]^ Some potential contaminants in the FW are presented in **Table** **11**, including chemical pollutants, heavy metals, microbes, and drug residues.

**Table 11:** Potential contaminants in FW used in hog feeding programs.

Contaminant type	Risk to pigs	Refs.
Chemical contaminants	Food waste may contain residues of agricultural chemicals such as pesticides, herbicides, and fungicides used during crop production. These chemicals can accumulate in food waste and pose health risks to pigs if consumed in high concentrations. It's essential to source FW from sources that adhere to safe agricultural practices and minimize the use of chemical inputs.	[156]
Heavy metals	Food waste from contaminated soils or environments may contain heavy metals such as lead, cadmium, and mercury. These metals can accumulate in animal tissues over time and pose risks to pig health and human food safety if ingested. Monitoring the sources of food waste and conducting soil and water testing can help identify potential heavy metal contamination.	[157]
Microbial pathogens	Food waste, especially meat and dairy products, can harbor pathogenic bacteria such as *Salmonella*, *Escherichia coli*, and *Listeria monocytogens*. These pathogens can cause foodborne illness in pigs and humans if not adequately controlled. Implementing proper sanitation and hygiene practices during food waste collection, storage, and feeding can help minimize the risk of microbial contamination.	[158]
Mycotoxins	Moldy or spoiled FW may contain mycotoxins produced by certain molds and fungi. Mycotoxins such as aflatoxins, ochratoxins, and fumonisins can harm pigs and cause adverse health effects, including liver damage, immune suppression, and reduced growth performance. Proper storage and handling of FW to prevent mold growth and mycotoxin formation are essential to prevent health complications.	[159]
Drug residues	Pharmaceutical or veterinary drug residues in FW can threaten pig health and human food safety. The residues of antibiotics, hormones, and other medications used in food production may accumulate in animal tissues and affect animal health and product quality. It's essential to avoid feeding FW containing prohibited substances and adhere to withdrawal for medicated feeds.	[160]
Physical contaminants	Food waste may contain physical contaminants such as plastic fragments, glass shards, metal pieces, or other foreign objects. These contaminants can cause injury or digestive issues if ingested by pigs. Thorough inspection and sorting of FW before feeding can help remove physical pollutants.	[161]

To mitigate the risks associated with contaminants in FW, it is essential to implement strict quality control measures. Ensuring the safety of food waste (FW) as animal feed requires a comprehensive approach that integrates several key measures. Proper processing of FW is critical to reduce contaminants and enhance safety.^[^ ^143^, ^162^ ^]^ Continuous monitoring through regular inspections and appropriate testing helps detect potential contamination before the waste is used as feed.^[^ ^17^, ^138^ ^]^ Maintaining detailed records of FW sources, handling procedures, and testing outcomes further supports traceability and accountability in feed production.^[^ ^22^, ^163^ ^]^ Finally, strict adherence to regulatory requirements and established guidelines governing food safety and animal feed production ensures compliance with legal and industry standards.^[^ ^40^, ^164^ ^]^

### 5.6 Using Organic Food Waste

Prioritize organic FW whenever possible, as it tends to have fewer chemical residues and is more environmentally sustainable. Organic waste from fruits, vegetables, grains, and legumes can be an excellent source of nutrients for pigs. However, ensure that organic waste is free from pesticide residues and other contaminants. FW from organic‐certified sources, such as organic farms, grocery stores, and restaurants, can be safely fed to livestock. Organic FW typically consists of organic fruits, vegetables, grains, and dairy products that meet organic standards and regulations. Byproducts generated during food processing, such as potato peels, apple pomace, and soybean hulls, can be repurposed as animal feed.^[^ ^17^ ^]^ These waste materials are often rich in fiber and nutrients and can be a valuable addition to livestock diets. Organic meat trimmings, bones, and seafood byproducts can be included in animal feed to provide additional protein and minerals. These byproducts are rich in nutrients and can help meet the dietary needs of livestock.

### 5.7 Quantity and Consistency of Food Waste

The quantity and consistency of food waste (FW) available from different sources play a significant role in determining its suitability as a component of swine diets. Consistent availability of sufficient quantities is particularly relevant in ensuring that nutritional requirements are met across different physiological stages of pig growth. Establishing dependable partnerships with food waste generators may help maintain a steady feedstock supply. According to Fung et al. (2019), variations in FW used for hog feed depend on several factors, including its nutrient composition, pigs’ dietary needs, and the availability of complementary feed ingredients. Evaluating the nutritional composition of FW can help determine its appropriateness for integration into pig diets. FW must support balanced nutrition in terms of energy, protein, vitamins, and minerals. Aspects such as moisture content, protein levels, and energy density are essential when estimating potential feeding rates. For instance, excessively wet feed may limit intake and lead to slower weight gains due to reduced dry matter consumption.^[^ ^165^ ^]^ Collaboration with animal nutrition professionals can be valuable in formulating diets that integrate FW effectively.^[^ ^143^ ^]^ Feeding strategies should be based on the characteristics of the FW and adjusted in line with the pigs’ specific nutritional demands.^[^ ^96^ ^]^

Variation in feed quantity is also influenced by the age, body weight, growth rate, and activity levels of the animals.^[^ ^166^ ^]^ Introducing FW gradually and observing the animals’ adaptation is one way to facilitate a smooth dietary transition. Ongoing assessment of growth, performance, and general health indicators can inform necessary changes in feeding levels.^[^ ^167^ ^]^ Ensuring consistency in the types and amounts of FW incorporated into pig diets may help reduce digestive upsets and promote stable nutrient intake.^[^ ^168^ ^]^ Diversity in FW sources can contribute to a more comprehensive nutrient profile and may enhance palatability. Periodic rotation of FW types can give pigs a broader range of nutrients and dietary experiences. Regularly monitoring feed intake, animal performance, and health remains essential. Formulation adjustments may be needed as conditions or animal requirements change over time^[^ ^87^, ^169^ ^]^ emphasize the importance of aligning feed quantities with parameters such as age, weight, breed, growth stage, activity level, and environmental conditions. A summary of general feeding estimates is presented in **Table** **12**. Attention to food safety and compliance with animal feed regulations is essential when FW is included in feeding programs. This includes record‐keeping of FW types and volumes and adherence to any applicable food safety or animal health guidelines. While FW presents a sustainable and potentially cost‐effective feed alternative, its successful use depends on thoughtful planning and consistent nutritional management.

**Table 12:** General guideline to help estimate the quantity of food waste needed for hog feeding operations.

Feeding guidelines	Implementation notes	Refs.
Calculate daily feed requirements	Start by calculating the daily feed requirement for each hog. This can be based on their physiological stage, weight, expected growth rate, and nutritional needs. A standard method is to provide ≈2–3% of the hogs' body weight in feed per day.	[96, 170]
Adjust for physiological stage	The feed requirements of hogs vary depending on their physiological stage. Young pigs (piglets) and growing pigs require more feed relative to their body weight than mature pigs (breeding or finishing hogs). Adjust the feed's quantity and quality based on the hogs' physiological stage.	[213, 214]
Consider nutritional needs	Ensure that the feed meets the nutritional requirements of hogs. This includes adequate levels of proteins, carbohydrates, fats, vitamins, and minerals. Consult with a veterinarian or nutritionist to develop a balanced diet that meets the specific nutritional needs of the hogs.	[96, 214]
Monitor body condition	Monitor the body condition of the hogs regularly to assess whether they are receiving the correct quantity of feed. Hogs should maintain a healthy body condition score, neither thin nor fat. Adjust the feed quantity accordingly based on changes in body condition.	[171, 211]
Adjust for activity level	Hogs that are more active (free range, on pasture) or in stages such as breeding, gestation, or lactation may require additional feed to meet their energy needs. Consider the activity level of the hogs when determining the feed quantity.	[19, 94]
Environmental factors	Environmental factors such as temperature, humidity, and housing conditions can affect the food intake of hogs. During extreme weather conditions, hogs' diets may require adjustments (either in quantity or content) to maintain their body temperature and energy levels.	[94, 172, 211]
Feed efficiency	Monitor feed efficiency (the amount of feed required to produce a unit of weight gain) to optimize feed usage and minimize feed wastage. Adjust feed quantities based on feed intake to ensure optimal growth and performance.	[173, 174]
Feed quality	Ensure the feed is of the highest quality and free from contaminants or spoilage. Poor quality can lead to reduced feed intake, digestive issues, and health problems.	[175, 176]
Regular monitoring and adjustment	Regularly monitor the hogs’ feed intake, growth performance, and overall health. Adjust feed quantities as needed based on changes in the hogs’ requirements and performance.	[177, 178]

## 6 Regulatory Considerations for Safe Food Waste Recycling in Swine Production

**Figure 6:**
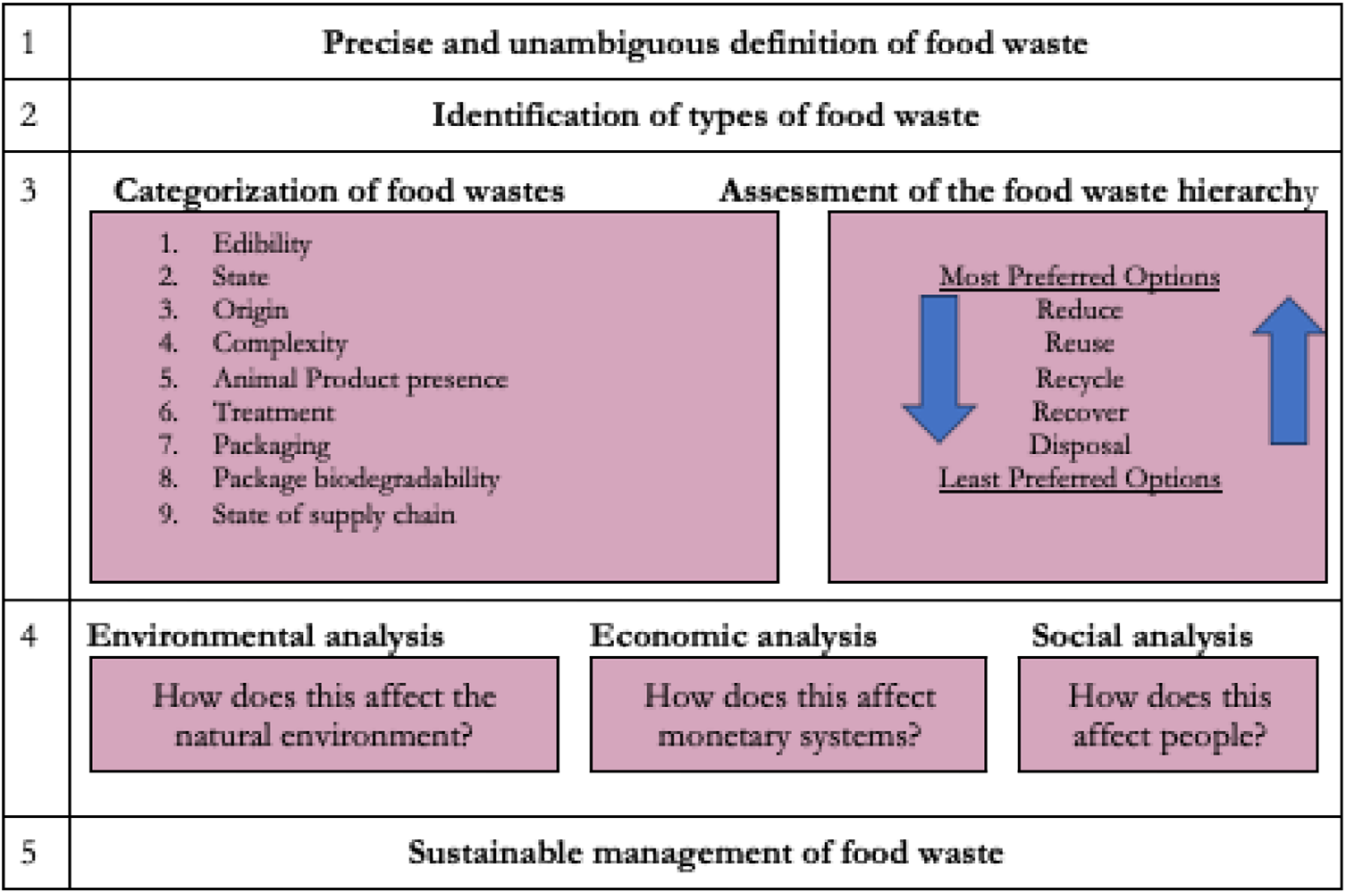
Integrating food waste management criteria into circular systems. Source: Adapted from ref. [40].

Pigs offer an efficient solution for repurposing food waste no longer suitable for human consumption. While they should not be fed rancid or moldy foods contaminated with harmful pathogens,^[^ ^107^, ^179^ ^]^ they excel at recycling organic waste that would otherwise be discarded. This reduces the environmental legacy of FW and helps transform our linear waste management system into a more circular, sustainable model by reintegrating nutrients back into the food chain and environment (**Figure** **6**). Nine criteria drive circular systems for FW management. Safe, nutrient‐rich FW flows into recycling (e.g., animal feed, composting), while hazardous or complex FW (e.g., moldy, non‐biodegradable packaging) is diverted from circular loops. Outcomes align with environmental resilience, economic efficiency, and social equity. The challenges we face with this endeavor revolve around safety and efficiency relevant to achieving sustainability and providing the nutrients required for each physiological stage. Pigs can become sick from food gone bad, just like people, so pasteurizing collected waste is imperative to ensure the animal's safety in this reclamation process. Implementing Hazard Analysis and Critical Control Points ensures FW products are safe and healthy with principles that help identify and control potential hazards in the processing of FW.^[^ ^180^, ^181^ ^]^ Regular monitoring and testing for contaminants such as pathogens, heavy metals, and toxins are essential to maintain feed safety. Feeding FW to pigs involves several regulatory considerations to ensure food safety, animal health, and environmental protection (**Table** **13**).

**Table 13:** Regulatory compliance necessary to feed FW as animal feed.

Regulatory area	Compliance requirement	Refs.
Food safety regulations	Food waste used as pig feed must meet food safety standards to prevent the transmission of diseases and contaminants to animals and humans. Regulations may specify requirements for the types of FW allowed, storage and handling practices, and permissible levels of contaminants.	[18]
Feed safety regulations	Many countries have regulations governing the production and use of animal feed, including feed ingredients such as FW. These regulations may include requirements for feed labeling, registration, composition, and quality control measures to ensure the safety and nutritional adequacy of the feed.	[182, 183]
Biosecurity measures	Biosecurity measures are essential to prevent the introduction and spread of diseases on farms. When feeding FW to pigs, biosecurity protocols should be implemented to minimize the risk of disease transmission. This may include screening FW for pathogens, controlling access to feed storage areas, managing wildlife and pests, and practicing good hygiene and sanitation measures.	[184]
Animal health regulations	Regulations related to animal health may govern the feeding of certain types of FW to pigs, mainly if there is a risk of introducing infectious agents or toxins that could harm animal health. Compliance with these regulations may involve risk assessments, veterinary oversight, and animal health and performance monitoring.	[185, 186]
Environmental regulations	Feeding FW to pigs can have environmental implications, such as nutrient management and waste disposal. Compliance with environmental regulations may involve proper management of manure and waste streams, nutrient management planning, and adherence to rules governing land application of manure.	[81]
Waste management regulations	Waste management regulations may apply to the collection, transportation, and disposal of FW generated by food processing facilities, restaurants, and other businesses. Compliance with these regulations may involve obtaining permits, following waste handling procedures, and ensuring proper disposal or recycling of FW.	[128]
Record‐keeping requirements	Regulatory compliance often requires maintaining detailed records of feed ingredients, feeding practices, and animal health monitoring. Accurate record‐keeping is essential for demonstrating compliance with regulations, traceability of feed ingredients, and responding to regulatory inquiries or audits.	[187]
Import and export regulations	If FW is imported or exported as pig feed, compliance with import and export regulations may be required. This may include obtaining permits, complying with import/export restrictions, and meeting phytosanitary or sanitary requirements.	[188]

Farmers should ensure that the FW collection and feeding practices comply with local regulations and guidelines. Familiarize yourself with legal requirements for waste management, animal feeding, and biosecurity. Obtain necessary permits and permissions before initiating FW feeding programs. Farmers and feed manufacturers should stay informed about applicable regulations, work closely with regulatory authorities and veterinary professionals, implement appropriate risk management practices, and maintain accurate feed production and usage records to ensure compliance with regulatory requirements when feeding FW to pigs. Establish an effective pest and rodent control program. Additionally, ongoing monitoring and review of regulatory changes and updates are essential to maintain ongoing compliance.

## 7 Economic and Environmental Benefits of Feeding Food Waste to Pigs

Food waste represents a significant environmental and economic burden, with millions of tons discarded annually.^[^ ^37^, ^189^, ^190^ ^]^ Redirecting this waste stream to pig feed mitigates waste management issues and reduces reliance on conventional feed crops.^[^ ^17^, ^62^ ^]^ This practice promotes a circular economy and enhances the sustainability of both the food and livestock industries. Repurposing FW for pig consumption is a sustainable approach to managing FW while providing an economical feed source for swine production.^[^ ^18^ ^]^ Feeding swine accounts for ≈80% of the costs required to produce pork products worldwide.^[^ ^41^ ^]^ The current expensive feed practice consists of crops like corn and soy being grown, harvested, dried, and transported to feed primary consumers, such as swine, which are being raised to feed humans at the top of the food chain. In this, most of the energy produced by crops is lost to the pigs, and humans reap a minimal yield of the caloric sum required to raise swine, despite the large amount of energy that is invested in farming. This issue can be curbed by altering how consumers and producers manage calorically dense FW. Once proper systems are in place, the energy and resources demanded by pig production will be a fraction of the current demand, and another vast issue–FW–will become an input, promoting a circular economy.

**Figure 7:**
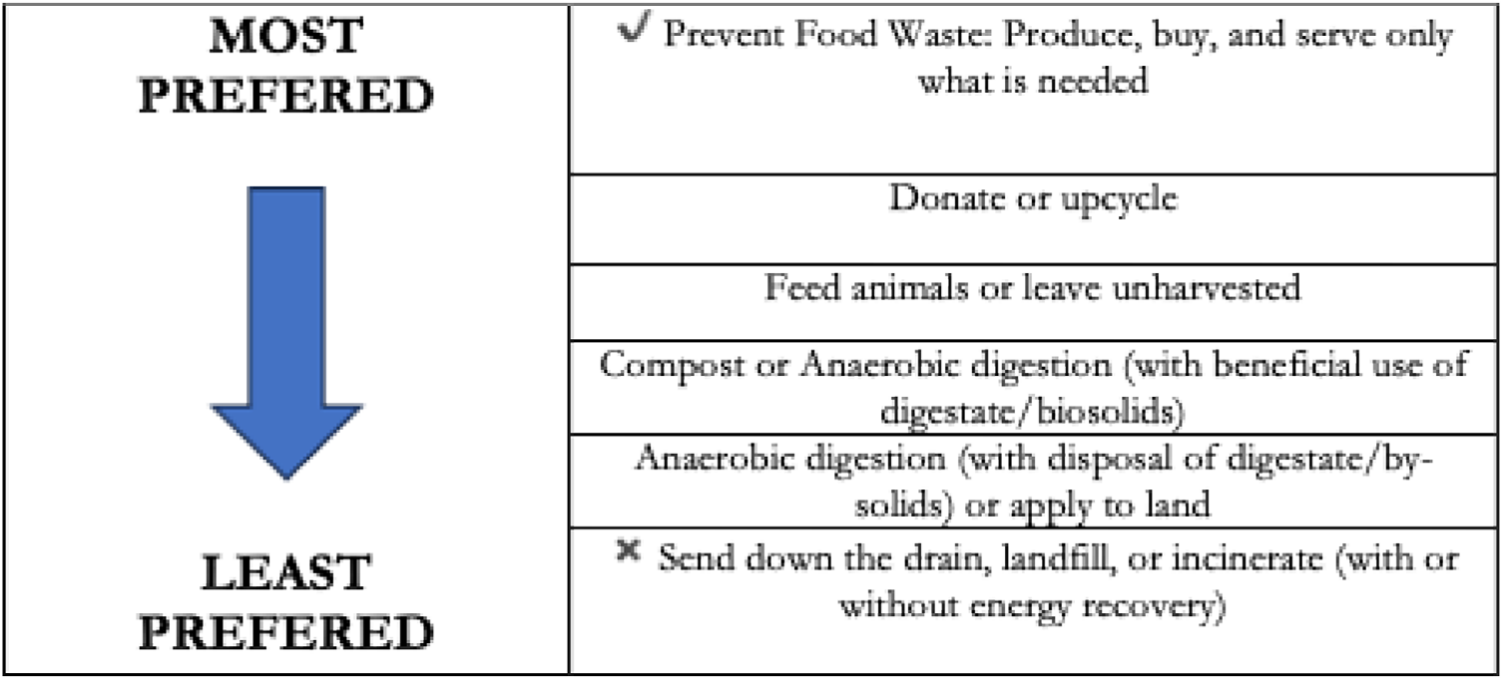
Food waste recycling priority scale. Source: Adapted from EPA.^[^ ^11^ ^]^

The environmental impact of using FW for compost or anaerobic digestion is far higher than the impact of using it to feed animals. However, all three options are better than sending waste to landfills. Various gases are emitted from organic matter through decomposition and feeding animals, and FW reduces the environmental impact (**Figure** **7**).

FW collection must first be separated and consolidated at the disposal site to gather the materials needed.^[^ ^191^ ^]^ This will be possible by partnering with local food distributors like restaurants and grocery stores and providing them with sanitary collection vessels, requiring that all labels and plastic must be separated upon disposal, which, if done correctly, will not incur a significant labor cost.^[^ ^139^ ^]^ These vessels will be collected on a set schedule and brought to the processing destination. FW will first be screened for remaining impurities and non‐organic materials and then passed into a large container for pasteurization.^[^ ^133^, ^192^, ^193^ ^]^ Last, the food must be rendered down to a dry state to make it shelf stable by evaporating its liquid content with extremely hot or cold temperatures.^[^ ^142^, ^194^ ^]^ All of this can be done by hand or with specialized equipment and requires routine sanitation procedures to reduce the risk of foodborne illness. From start to finish, this process requires multiple waste collection vessels, vehicles for transporting those vessels, and a property with a warehouse to pasteurize and render the food waste.

Specific feeding equipment and methods are required for farmers to efficiently supply livestock with animal feed (**Table** **14**). Efficient feed management is central to the sustainability of pig production systems, as feed constitutes a significant portion of production costs and resource use (Table 14). The integration of advanced feeding equipment not only optimizes nutrient delivery and minimizes feed wastage but also supports animal health, improves growth performance, and reduces environmental impacts associated with nutrient excretion and resource overuse.

**Table 14:** Technological innovations in feeding equipment for sustainable pig production.

Equipment	Function and sustainability benefits	Refs.
Automated feeders	Automated feeders precisely dispense feed based on the pigs' consumption patterns, reducing waste and ensuring consistent nutrient intake. These systems can be programmed to deliver specific amounts of feed at scheduled times, optimizing feed efficiency.	[195]
Wet‐dry feeders	Wet‐dry feeders provide pigs with feed and water in the same unit, reducing waste by preventing feed spillage and contamination. These feeders promote better feed intake and can improve feed conversion ratios.	[196]
Phase feeding systems	Phase feeding systems adjust the nutritional content of the feed according to the pigs' growth stages. This tailored approach reduces excess nutrient excretion and enhances feed efficiency, contributing to sustainable farming practices.	[197, 198]
Feed management software	Advanced feed management software helps farmers monitor and control feed distribution, track inventory, and analyze consumption patterns. This technology aids in optimizing feed use and minimizing waste.	[199]

Furthermore, technological innovations such as PLF and genetic improvements are driving significant sustainability gains in modern pig farming. PLF technologies, including sensors, automated monitoring systems, and data analytics, enable farmers to monitor and manage pigs' health, growth, and feed intake in real‐time, facilitating early disease detection, efficient feed allocation, and improved resource management.^[^ ^50^, ^200^ ^]^ Simultaneously, genetic improvement programs focus on enhancing feed efficiency, disease resistance, and growth rates, contributing to more sustainable production systems. Advances in genetic engineering and genomics offer opportunities to develop pigs that require less feed and produce less waste, thereby reducing environmental impacts and increasing overall farm efficiency.^[^ ^201^, ^202^ ^]^

## 8 Food Waste Start‐Up: Business Model for Adopting the Piggy Solution

In recent years, sustainable business practices have become increasingly crucial in addressing environmental challenges while ensuring economic viability (b).^[^ ^62^ ^]^ The Piggy Solution can be designed to recover phosphorus and other nutrients from the diverse FW streams. This closed‐loop approach addresses environmental concerns, especially those related to FW's greenhouse emissions, and creates economic opportunities through resource recovery and product development.^[^ ^81^, ^203^ ^]^ Business analysis of “Piggy Solution” should include ‐

### 8.1 Customer Segments

Primary customers include pig farmers, livestock feed manufacturers, food processing industries generating FW, and agricultural cooperatives. These segments benefit from reduced feed costs, enhanced nutrient availability, and compliance with sustainable farming practices.^[^ ^204^ ^]^

### 8.2 Value Proposition

The conversion of FW into pig feed offers a compelling value proposition: environmental sustainability by reducing FW and resource efficiency through recycling and economic profitability by lowering feed production costs and creating market opportunities for sustainable products.^[^ ^205^, ^206^ ^]^

### 8.3 Channels

Distribution channels involve partnerships with food processors for waste collection, direct sales to pig farmers and feed manufacturers, and collaborations with agricultural extension services for education and promotion.^[^ ^52^ ^]^

### 8.4 Revenue Streams

Revenue streams include sales of processed pig feed, service contracts for waste collection and processing, and potential partnerships with food industries for waste supply agreements.^[^ ^139^ ^]^

### 8.5 Key Resources, Activities, and Partnerships

Critical resources will include FW collection infrastructure, processing technology for feed production, partnerships with food industries for waste sourcing, and access to agricultural markets for product distribution. Core activities should encompass FW collection, processing into feed ingredients, formulation and production of balanced pig feed, quality control and assurance, regulatory compliance management, and stakeholder education on sustainable feed practices. Partnerships with food processors, technology providers for feed processing, agricultural cooperatives, and government agencies are essential for scaling operations, regulatory compliance, and market penetration.^[^ ^207^, ^208^ ^]^

### 8.6 Cost Structure

Costs include operational expenses for food waste collection and processing, feed production and distribution, investments in research and development for technological innovation, and compliance with regulatory standards.^[^ ^62^ ^]^ Additionally, scalability challenges such as transportation logistics, infrastructure needs, and labor for sorting must be considered, particularly for small‐scale farms. To address these, decentralized processing hubs and cooperative models may offer cost‐effective and practical solutions.

Implementing FW conversion into pig feed faces challenges such as sourcing consistent and quality FW, ensuring food safety and nutritional adequacy, navigating regulatory frameworks, and building market acceptance. Sustainable strategies like developing robust supply chain partnerships, investing in technology for efficient waste conversion, conducting rigorous testing and certification processes, and engaging stakeholders through education and advocacy will help overcome some of these challenges. Also, farmers can access training through agricultural extension services, which provide region‐specific guidance on food waste sourcing, safety protocols, and feed formulation. Additionally, digital tools such as mobile applications like MySusCoff^[^ ^209^ ^]^ and FAO's FLAPP (FAO n.d.), online training modules,^[^ ^210^ ^]^ and platforms like the FAO's Technical Platform on the Measurement and Reduction of Food Loss and Waste^[^ ^216^ ^]^ offer accessible resources for improving technical know‐how. These platforms help disseminate best practices, connect producers to regulatory information, and foster peer‐to‐peer learning, thereby strengthening the adoption of food‐waste‐to‐feed systems across diverse agricultural contexts. The conversion of FW into pig feed presents a viable business model contributing to environmental sustainability, economic efficiency, and food security. By repurposing FW into valuable agricultural inputs, stakeholders can achieve both ecological benefits and economic gains, fostering a more sustainable and resilient farming system.

## 9 Conclusion

Feeding FW to swine provides a promising potential avenue for utilizing FW as a resource in hog farming practices. This review underscores the importance of addressing FW challenges while enhancing sustainability within the hog farming industry. By repurposing FW as a feed source for pigs, farmers can achieve multiple benefits, including reducing waste sent to landfills, lowering feed costs, and improving environmental sustainability. Furthermore, feeding FW to pigs offers a practical solution to food security concerns by maximizing resource efficiency and promoting circular economy principles. However, successful implementation of FW utilization in hog farming requires careful consideration of regulatory compliance, nutritional balance, and food safety concerns. Collaboration between farmers, policymakers, researchers, and waste management stakeholders is essential to develop effective strategies and guidelines for safe and sustainable FW utilization in hog feed. Such collaboration has yielded benefits in South Korea as the volume‐based food waste charging scheme has significantly reduced household food waste by incentivizing waste minimization and encouraging segregation and reuse, making it a model for effective policy‐driven intervention. Harnessing FW for sustainable hog farming offers a viable solution to the intertwined challenges of waste management, environmental sustainability, and agricultural efficiency. Repurposing FW into nutritious pig feed can help significantly reduce waste directed to landfills, thereby cutting greenhouse gas emissions and conserving valuable landfill space. This practice contributes to a circular economy and enhances the economic viability of pig farming by lowering feed costs and providing a reliable source of nutrients for swine. However, implementing this solution requires meticulous safety and regulatory compliance, ensuring the feed is free from contaminants and nutritionally adequate for the animals. Advanced processing techniques such as heat treatment, fermentation, and pelleting are critical in transforming FW into a safe and digestible form for pigs. Moreover, fostering collaboration among FW generators, processors, farmers, and regulatory bodies is essential to establishing an efficient and sustainable food waste‐to‐feed system. Continued research and development in this area should focus on optimizing the nutritional profiles of food waste‐based feeds, mitigating risks such as mycotoxin contamination, enhancing scalable processing technologies, and expanding the range of safe and acceptable waste materials for diverse farming systems. Education and awareness campaigns can also significantly promote source separation and reduce contamination, thereby improving the quality of FW collected for pig feed.

Implementing sustainable feeding strategies requires an initial investment, but the long‐term benefits often outweigh the costs. Conducting a cost‐benefit analysis can help farmers understand the economic advantages of adopting sustainable practices, such as reduced feed costs, improved animal health, and higher productivity. Educating farmers about sustainable feeding practices and providing training on new technologies and techniques are crucial for successful implementation. Extension services, workshops, and online resources can support farmers in adopting and maintaining sustainable practices. Government policies and regulations play a vital role in promoting sustainable pig farming. Incentives, subsidies, and support programs can encourage farmers to adopt sustainable feeding strategies. Additionally, regulations on feed ingredients, waste management, and animal welfare standards ensure that sustainability goals are met.

## Conflict of Interest

The authors declare no conflicts of interest.

## Author Contributions

M.C.O. and C.E.H. conceived and designed the work, conducted a literature review to collect and analyze pertinent information. M.C.O. and C.E.H. wrote the first draft, and all the authors contributed to subsequent drafts. S.P. supervised the study and contributed to the final draft.
